# Study of MicroRNAs Related to the Liver Regeneration of the Whitespotted Bamboo Shark, *Chiloscyllium plagiosum*

**DOI:** 10.1155/2013/795676

**Published:** 2013-09-17

**Authors:** Conger Lu, Jie Zhang, Zuoming Nie, Jian Chen, Wenping Zhang, Xiaoyuan Ren, Wei Yu, Lili Liu, Caiying Jiang, Yaozhou Zhang, Jiangfeng Guo, Wutong Wu, Jianhong Shu, Zhengbing Lv

**Affiliations:** Institute of Biochemistry, College of Life Sciences, Zhejiang Sci-Tech University, Hangzhou 310018, China

## Abstract

To understand the mechanisms of liver regeneration better to promote research examining liver diseases and marine biology, normal and regenerative liver tissues of *Chiloscyllium plagiosum* were harvested 0 h and 24 h after partial hepatectomy (PH) and used to isolate small RNAs for miRNA sequencing. In total, 91 known miRNAs and 166 putative candidate (PC) miRNAs were identified for the first time in *Chiloscyllium plagiosum*. Through target prediction and GO analysis, 46 of 91 known miRNAs were screened specially for cellular proliferation and growth. Differential expression levels of three miRNAs (xtr-miR-125b, fru-miR-204, and hsa-miR-142-3p_R-1) related to cellular proliferation and apoptosis were measured in normal and regenerating liver tissues at 0 h, 6 h, 12 h, and 24 h using real-time PCR. The expression of these miRNAs showed a rising trend in regenerative liver tissues at 6 h and 12 h but exhibited a downward trend compared to normal levels at 24 h. Differentially expressed genes were screened in normal and regenerating liver tissues at 24 h by DDRT-PCR, and ten sequences were identified. This study provided information regarding the function of genes related to liver regeneration, deepened the understanding of mechanisms of liver regeneration, and resulted in the addition of a significant number of novel miRNAs sequences to GenBank.

## 1. Introduction

Cartilaginous fish are jawed vertebrates that diverged from the common ancestor of humans and teleost fish approximately 530 million years ago [1]. Similar to teleost fish, cartilaginous fishes possess complex physiological systems, such as an adaptive immune system and a pressurized circulatory system [2]. The white spotted bamboo shark (*Chiloscyllium plagiosum*) is a cartilaginous fish that is widely distributed in cold seas along the coasts of the eastern Pacific. Economically, this shark is one of the most important marine animals, possessing both scientific and commercial food values. The liver of *C. plagiosum*, accounting for 75% of the weight of the viscera, possesses immune regulatory functions and contains bioactive substances. Several investigators have isolated and cloned stimulatory factors related to liver regeneration and immune regulation from *Chiloscyllium plagiosum* [3–6], but there have not yet been miRNAs reported for this species.

MicroRNAs (miRNAs) are a family of small, noncoding, and single-stranded RNAs that consist of approximately 19–25 nucleotides derived from the stem regions of hairpin transcripts (referred to as “pre-miRNAs”). These small RNAs act as regulators, leading to either mRNA cleavage or translational repression by complementarily hybridization to the 3′-untranslated regions (3′ UTRs) of their target mRNAs. The formation of mature miRNAs involves several steps. First, most miRNAs are transcribed as long primary transcripts (pri-miRNAs) by RNA Pol II, although a minor group of miRNAs associated with Alu repeats can be transcribed by RNA Pol III. The primary transcript is processed by the RNase III enzyme Drosha in the nucleus to give one or more 60–100 nt long hairpin precursor sequences (pre-miRNAs). Together with DiGeorge syndrome critical region gene 8 (DGCR8), Drosha forms a large complex known as the Microprocessor complex; DGCR8 interacts with pri-miRNAs and assists Drosha in cleaving the substrate. The hairpin precursor is then exported by Exportion-5 to the cytoplasm, where the mature miRNA is excised by another RNase III enzyme, Dicer. Following Dicer cleavage, the resulting *≈*22 nt RNA duplex is loaded into the Ago protein to create the effector complex, RISC. One strand of the *≈*22 nt RNA duplex remains with Ago as a mature miRNA and targets the 3′ UTR complementary pairing area of the target gene to approximately 2–8 bases of the 5′ end of the miRNA [7–9]. Negative posttranscriptional regulation by miRNAs has been observed in various biological processes, including viral disease [10], lipid metabolism [11], and cellular proliferation and differentiation [12].

Research into the mechanisms of liver cell growth and apoptosis has focused on individual signal molecules, important transcription factors, and related target genes and signaling pathways. Research has been less comprehensive, however, regarding the more analytical aspects of gene expression regulation. A substantial body of evidence has indicated that miRNAs are abundant in the liver and play an important role in liver development, disease, and regeneration [13–15]. In this study, we carried out deep sequencing in combination with bioinformatics and genomic sequencing to identify miRNAs in *Chiloscyllium plagiosum* liver. Although sequencing of *Chiloscyllium plagiosum* genome has not yet been completed, the *Callorhinchus milii* genome was recently proposed to be a model for cartilaginous fish genomes [16]. Therefore, we used the *Callorhinchus milii* genome to predict pre-miRNA hairpin structures. In this study, we characterized small RNAs and identified miRNAs in the *C. plagiosum* liver. We further analyzed miRNA clusters and the expression of *Chiloscyllium plagiosum* miRNAs. Our results may contribute to liver miRNA research not only for fish but also for other species. This is the first comprehensive report about the miRNA of cartilaginous fish and adds several novel miRNAs to the database, which will help to identify homologs in other species. In addition, screening differentially expressed genes in normal and regenerating liver tissues by DDRT-PCR will provide valuable information for further study of the newly discovered functional genes related to liver regeneration and the relationship between differentially expressed genes and miRNAs. We have laid the foundation for further understanding of the function of miRNAs in liver regulation in *Chiloscyllium plagiosum*. 

## 2. Materials and Methods

### 2.1. Sample Preparation and Liver Excision

A live *Chiloscyllium plagiosum* was purchased from a Guangdong aquatic product market. Two-thirds of the liver was surgically removed, using a 2% chloral hydrate solution (300 mg per Kg weight of shark) as an anesthetic. The wound was then sutured, and the shark was placed into seawater to continue feeding. Regenerating liver tissues were obtained 0 h and 24 h after the partial hepatectomy (PH) by opening the suture and removing the cut border of the left liver. These experiments complied with the relevant national animal welfare laws and were conducted under guidelines approved by the China Wildlife Conservation Association.

### 2.2. Small RNA Library Construction and Deep Sequencing

The total RNA of the liver was extracted using the mirVana miRNA Isolation Kit (Ambion, Austin, USA) according to the manufacturer's protocol. Small RNAs were size fractionated and ligated to the SRA 5′ adapter. This step was followed by SRA 3′ adapter ligation and size fractionation to isolate RNAs of 64–99 nt. The RNAs were then converted to single-stranded cDNA using Superscript II reverse transcriptase (Invitrogen, CA, USA) and Illumina's small RNA RT Primer. This pool was once again size fractionated to isolate cDNA in the 80–115 bp range that contained miRNAs. Next, the cDNA was PCR-amplified for 20 cycles using Illumina's small RNA primer set and Phusion polymerase (New England Lab, USA). The PCR products were size fractionated and recovered for sequencing on a Genome Analyzer GA-II (Illumina, San Diego, USA) following the vendor's recommended protocol for small RNA sequencing. Following real-time sequencing, image analysis and base calling by Illumina's Real-Time Analysis version 1.8.70 (RTA v1.8.70), raw sequencing reads were obtained using Illumina's Sequencing Control Studio software version 2.8 (SCS v2.8). The extracted sequencing reads were then used for standard data analysis. 

### 2.3. Standard Data Analysis

**Figure 1:**
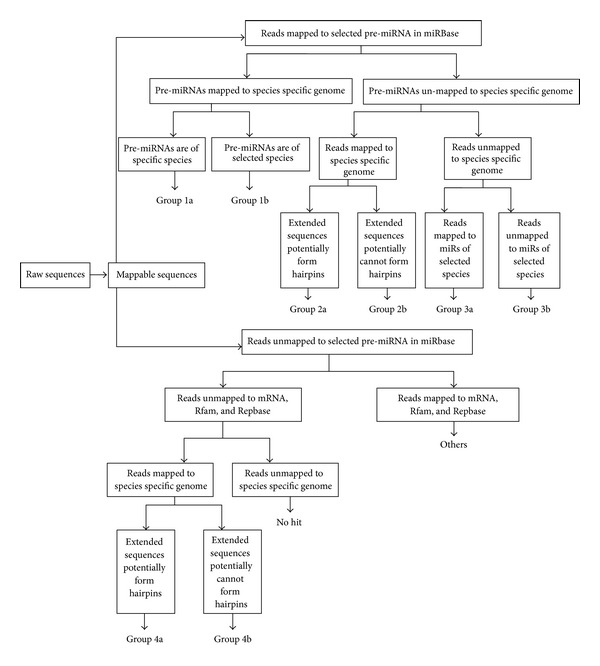
Procedure for mapping reads to database sequences. (1) specific species, that is, Callorhinchus milii; (2) selected species: *Fugu rubripes, Oryzias latipes, Xenopus laevis, Xenopus tropicalis, Homo sapiens,* and *Mus musculus. *

Following the removal of adapter sequences and low-quality reads, clean reads between 15 and 30 bases in length were processed for bioinformatic analysis. A proprietary pipeline script, ACGT101-miR v4.2 (LC Sciences) [17, 18], was used for the sequencing data analysis. Various “mappings” were performed with unique sequences against pre-miRNAs and mature miRNAs sequences from selected species (*Danio rerio, Fugu rubripes, Oryzias latipes, Xenopus laevis, Xenopus tropicalis, Homo sapiens*, and* Mus musculus*) listed in the miRBase v19.0 (http://www.mirbase.org/) as well as the genome of *Callorhinchus milii *(http://esharkgenome.imcb.a-star.edu.sg/resources.html). The mapping process is presented in Figure 1. Flanking sequences of mapped reads were subjected to secondary structure analysis to predict pre-miRNA sequences using Mfold software. The criteria used for miRNA annotation and hairpin structure determination are presented in Table 1 [19, 20].

### 2.4. Target Prediction for miRNAs and GO Analysis

We compared 1839 ESTs of* Chiloscyllium plagiosum* with 1344 ESTs with 98% similarity using the cd-hit-454 program [21, 22]. Ninety-one mature miRNA sequences were used as custom sequences to search ESTs and perform target prediction using the program miRanda v1.0b [23] with the following parameter settings: Gap Open Penalty: −8.0; Gap Extend:−2.0; Score Threshold: 50.0; Energy Threshold: −20.0  kcal/mol; Scaling Parameter: 2.0. The target sequences were annotated using BLAST to the GO database (http://www.geneontology.org/), thus predicting which processes the miRNAs participate in. 

### 2.5. Stem-Loop RT-PCR

Seven candidate miRNAs were chosen for verification of expression using stem-loop RT-PCR. Four PC miRNAs were randomly selected (PC-3p-186_7748, PC-5p-970_1302, PC-5p-108_13860, and PC-5p-14_74120), and 3 known miRNAs were also analyzed: fru-miR-204a, hsa-miR-142-3p_R-1, and fru-miR-126. Total RNA was extracted using Trizol reagent and reverse transcribed using a Reverse Transcription System (Promega). The 5 *μ*L reactions contained 250 ng of RNA sample and 2 *μ*M of stem-loop RT primer (0.5 *μ*L). The reactions were incubated for 45 min at 42°C and 5 min at 95°C and then held at 4°C. 20 *μ*L PCR reactions (2× Taq PCR Master Mix, Lifefeng) were run using 0.5 *μ*L of cDNA product by incubating for 5 min at 95°C, followed by 40 cycles of 94°C for 30 sec, 60°C for 30 sec, and 72°C for 30 sec. The PCR products were detected with gel electrophoresis. The positive control was 18s rRNA [4]. All of the primers that were used for RT-PCR are listed in Table 2. The reverse primers for PC-3p-186_7748 and fru-miR-204a were miR-Reverse1, and fru-miR-126 miR-Reverse3, respectively, while the remaining 4 miRNAs used miR-Reverse2.

### 2.6. Real-Time PCR Analysis

According to our literature search, the small RNA sequencing results, miRNA target gene results, and GO analysis, three known miRNAs (hsa-miR-142-3p_R-1, xtr-miR-125b, and fru-miR-204) related to liver regeneration and liver diseases were assayed in normal and regenerating liver tissue at 0 h, 6 h, 12 h, and 24 h using real-time PCR. A FastStart Universal SYBR Green Master (ROX) fluorescence quantitative PCR reaction kit (Roche) was used, and a 20 *μ*L reaction system was designed according to the kit instructions. Each sample was repeated 3 times, and the cycling conditions were as follows: 95°C for 2 min followed by 40 cycles of 95°C for 15 s and 60°C for 30 s. A melting curve analysis was used to ensure the specificity of real-time PCR primers.

### 2.7. DDRT-PCR

The total RNA of normal and regenerating liver tissues at 24 h was extracted using Trizol and was reverse transcribed using a Reverse Transcription System (Promega). The 25 *μ*L reactions contained 1250 ng of RNA and 50 *μ*M of anchor primer (2.5 *μ*L). The reactions were incubated for 45 min at 42°C and 5 min at 95°C and then held at 4°C. 10 *μ*L PCR reactions (2× Taq PCR Master Mix, Lifefeng) were performed using two anchor primers and twenty random primers. All of the primers used for DDRT-PCR are listed in Table 3. The 10 *μ*L reactions contained 5 *μ*L of 2× Taq PCR Master Mix, 0.1 *μ*L 50 *μ*mol/L anchor primer, 0.5 *μ*L 10 *μ*mol/L random primer, 0.5 *μ*L cDNA, and 3.9 *μ*L ddH_2_O. The reactions were incubated for 5 min at 95°C, followed by 40 cycles of 95°C for 30 sec, 40°C for 2 min, and 72°C for 1 min. The PCR products were detected by nondenaturing polyacrylamide gel electrophoresis and silver staining. 

### 2.8. Cloning and Sequencing

The target fragments were recovered using the crushing and soaking method. The screened differential genes were used as templates, and the corresponding anchor primers and random primers were used as primers for PCR. The PCR reaction system and procedure were the same as the previous ones. After assaying for and recovering the target differential fragments, they were cloned into the pMD18-T simple vector to construct recombinant plasmids. The differential genes were tested through bacterial PCR. The 10 *μ*L reactions contained 5 *μ*L 2× Taq PCR Master Mix, 0.4 *μ*L 10 *μ*mol/L PCR Forward Primer, 0.4 *μ*L 10 *μ*mol/L PCR Reverse Primer, 0.5 *μ*L cDNA and 3.9 *μ*L ddH_2_O. The reactions were incubated for 5 min at 95°C, followed by 40 cycles of 95°C for 30 sec, 40°C for 2 min, and 72°C for 1 min. DNA sequencing was performed by Shanghai Sunny Biotechnology Co., Ltd. The obtained target differential genes were matched to NCBI EST library sequences through BLAST.

## 3. Results

### 3.1. Screening miRNAs in *Chiloscyllium plagiosum* Liver

**Figure 2:**
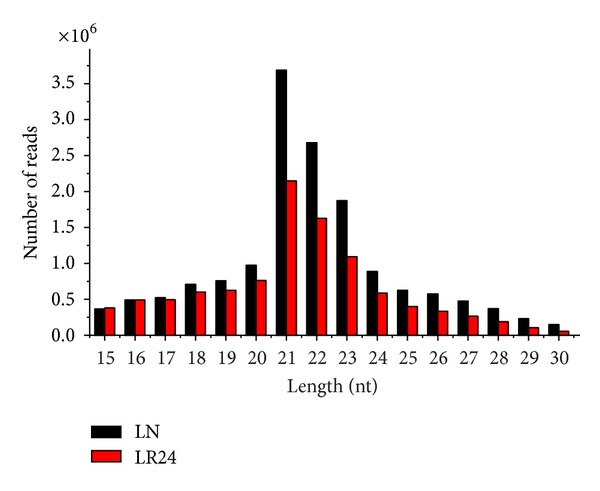
Length distribution of small 15-to-30 nucleotide RNAs in LN and LR24. The nucleotide (nt) lengths of the reads are indicated on the *x*-axis, and the number of total reads are indicated on the *y*-axis.

Raw reads in which the 3 adapters were not found and those with fewer than 15 bases after the 3 adapters were deleted; junk reads were removed. Following these changes, 15,361,801 and 10,146,583 reads remained in the *Chiloscyllium plagiosum* normal and regenerating livers at 0 h and 24 h after PH. The length distribution of the small RNA reads ranged from 15 to 30 nt (Figure 2). Through mapping analysis, 15,361,801 and 10,146,583 reads could be distributed into the groups that are presented in Table 4. 

**Figure 3:**
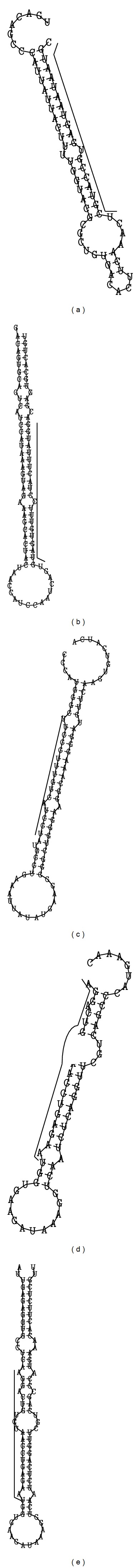
Secondary structures of 5 pre-miRNAs. (a) fru-miR-126, (b) hsa-miR-142-3p_R-1, (c) fru-miR-204a, (d) ola-miR-1388-3p_R+5/ola-miR-1388-5p_R-1, (e) PC-5p-14_74120/PC-3p-525510_1. The underlined letters refer to the candidate miRNA sequences. For (d) and (e), the underlined letters refer to the 5p miRNAs, and the upper-case letters refer to the 3p miRNA sequences.

Two hundred and fifty-seven miRNAs were identified in *Chiloscyllium plagiosum*, including 91 known miRNAs and 166 *Chiloscyllium plagiosum* putative candidate (PC) miRNAs. In addition, 31 PC miRNAs were found only in 24 h (after PH) regenerative liver tissue, and 49 PC miRNAs were found only in normal liver tissue. A total of 76 unique miRNA reads were mapped to the pre-miRNAs of selected species in the miRBase, and these pre-miRNAs were further mapped to the *Callorhinchus milii* genome (group 1b). Additionally, 15 miRNA reads mapped to pre-miRNAs of the selected species but not to the *Callorhinchus milii* genome. These 15 miRNA reads were mappable to the *Callorhinchus milii* genome, and the extended sequences could potentially form hairpins (group 2a). Additional 166 PC miRNAs that were identified did not map to any known pre-miRNAs, either in the mRNA, Rfam, or Repbase databases. These reads could map to the *Callorhinchus milii* genome, however, and exhibited the potential to form hairpin structures (group 4a). The predicted stem-loop secondary structures of 5 pre-miRNAs (fru-miR-126, hsa-miR-142-3p_R-1, ola-miR-1388-3p_R+5/ola-miR-1388-5p_R-1, PC-5p-14_74120/PC-3p-525510_1, and fru-miR-204a) from the Mfold database are presented in Figure 3. Information about all of these miRNAs is presented in supplementary Table 1; see in Supplementary Material available online at http://dx.doi.org/10.1155/2013/795676.

### 3.2. The Expression of *Chiloscyllium plagiosum* miRNAs

The average number of reads of known miRNAs in normal and regenerating liver tissues was 8173 and 8972, respectively. The average reads of PC miRNAs were 1687 and 1781, respectively. One hundred twenty-eight PC miRNA reads were less than 10 nt, which is consistent with a previous observation that nonconserved miRNAs are generally expressed at lower levels and represent tissue- or development-specific expression patterns [24]. Log_2_ ratios of 116 miRNAs reads in regenerating and normal tissues were larger than 1, of which only 5 miRNAs reads were more than 10 nt, and only 8 known miRNAs were included. Therefore, both miRNA expression type and abundance have no obvious differences during liver regeneration from 0 h to 24 h.

Moreover, miRNAs are often present in the genome in clusters, where several miRNAs are aligned in the same orientation and transcribed as a polycistronic message, allowing them to act cooperatively. Based on miRBase's definition of a miRNA cluster (10,000 bp), we discovered a total of 34 clusters in the *Callorhinchus milii *genome, of which 16 clusters were generated from the 16 pre-miRNAs, and 2 mature miRNAs were cleaved from each pre-miRNA (Supplementary Table 2). The largest cluster (no. 27) contained six miRNAs.

### 3.3. Target Predictions for miRNAs and GO Analysis

miRNAs are involved in a wide variety of biological processes by binding target sites with seed sequences [25, 26]. Identifying the total number of target sites, especially for those low-abundance and species-specific miRNAs, is helpful for appreciating the breadth of miRNA functions [27]. We calculated the potential binding sites between miRNAs and the ESTs of *Chiloscyllium plagiosum* with miRanda to determine the potential genes targeted by miRNAs. Of 91 known miRNAs, 86 miRNAs had targeted genes; most miRNAs have more than one, and 144 miRNAs had multiple predicted target sites. Most predicted targeted genes may be regulated by more than one miRNA. 

**Figure 4:**
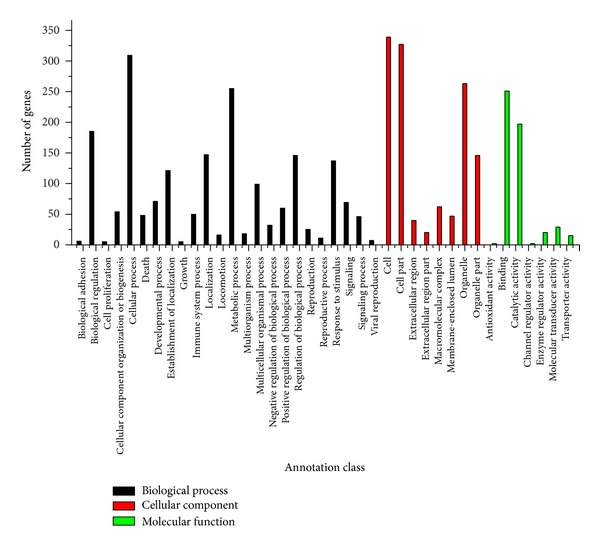
GO analysis of targeted genes. The classes are shown on the *x*-axis; the number of genes for each class is shown on the *y*-axis. The number of genes for the three classes—biological process, cellular component, and molecular function—is 687, 608, and 612, respectively.

**Figure 5:**
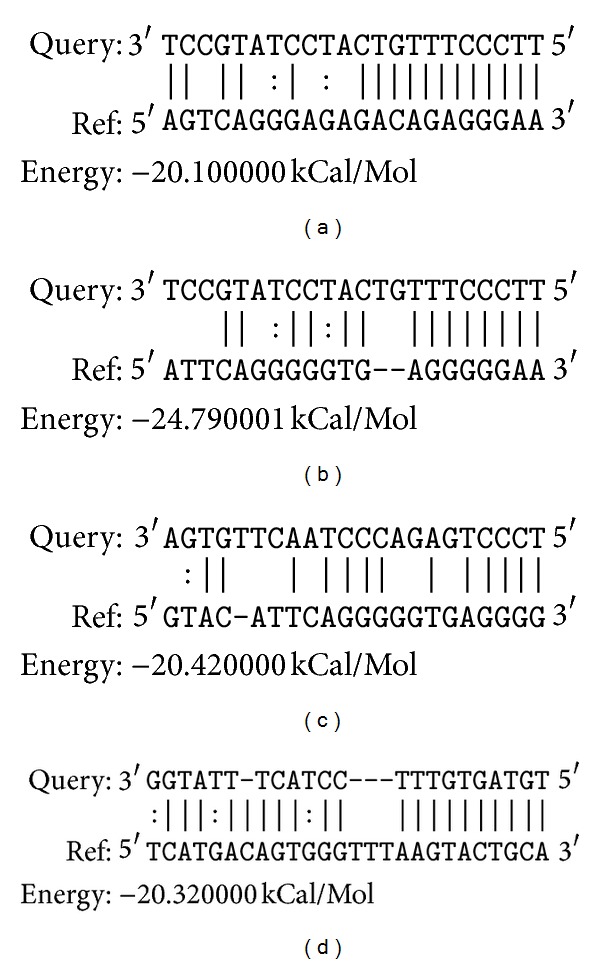
The potential binding sites between miRNAs and the ESTs of *Chiloscyllium plagiosum.* Query represents the miRNAs sequence, and Ref represents their predicted targets. (a) fru-miR-204; (b) fru-miR-204; (c) xtr-miR-125b; (d): has-miR-142-3p_R-1.

The functional classification for targeted genes was analyzed using Gene Ontology analysis. The targets that were functionally annotated in the GO database could be divided to 38 subclasses, presented in Figure 4. Forty-six miRNAs were related to cell proliferation and growth terms (Supplementary Table 3), including the following miRNAs: xtr-miR-34a_R-1 [28], fru-miR-122_R+1 [29], fru-miR-27b_R-1 [30], fru-miR-181b_R+1 [31], fru-miR-23b_R-1 [32], ola-miR-144_L-1R+2_1ss12TA [33], ola-miR-103_R+2 [29], and fru-miR-26 [34]. Based on comprehensive consideration of our literature search, the small RNA sequencing results, miRNA target gene results, and GO analysis, three known miRNAs (hsa-miR-142-3p_R-1, xtr-miR-125b, and fru-miR-204) related to liver regeneration and liver diseases were screened to the following experiments, and the potential binding sites between miRNAs and the ESTs of *Chiloscyllium plagiosum* with miRanda were presented in Figure 5. 

### 3.4. Real-Time PCR Analysis of the miRNAs of Different Liver Regenerative Stages

**Figure 6:**
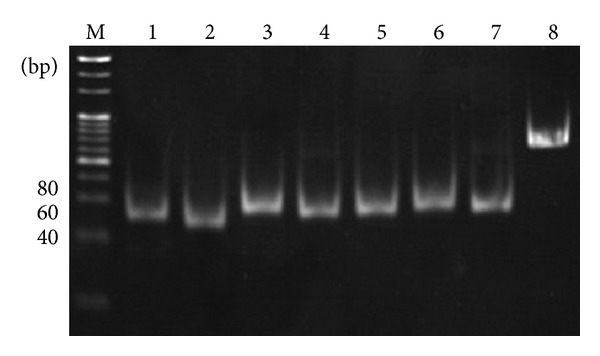
The identification of 7 miRNAs by stem-loop RT-PCR. The mRNA expression of 7 miRNAs was confirmed. The sizes of the PCR products were approximately 55 bp. 18s rRNA was used as a positive control. M: 20 bp DNA ladder maker; 1–12: PC-3p-186_7748, PC-5p-970_1302, PC-5p-108_13860, PC-5p-14_74120, hsa-miR-142-3p_R-1, fru-miR-204, fru-miR-126, 18s rRNA.

**Figure 7:**
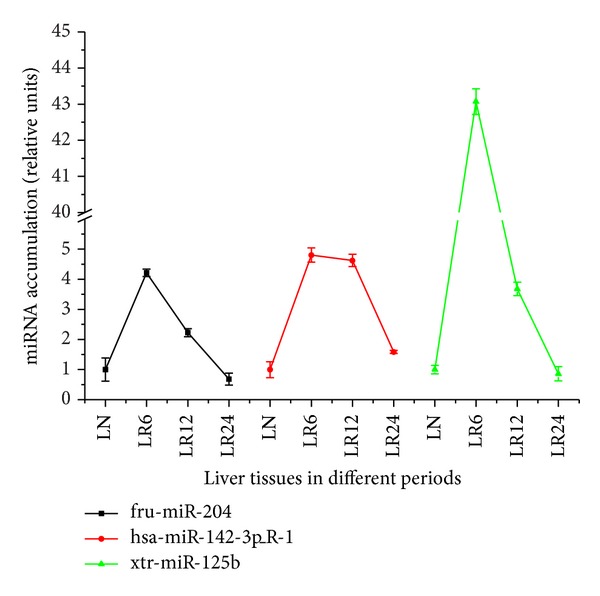
Relative expression changes of miRNAs in four periods of liver regeneration. The Real-time PCR results were processed by Microsoft Office Excel, and 2^−ΔΔ*C*_*t*_^ was used to represent the miRNA differential expression. Δ*C*_*t*_ = *C*_*t*_ (target gene) −  *C*_*t*_ (reference gene) ΔΔ*C*_*t*_ = *C*_*t*_ (experiment group) −  *C*_*t*_ (control group). the live issues in different periods are indicated on the *x*-axis, and the value of 2^−ΔΔ*C*_*t*_^ showed in Table 5 are indicated on the *y*-axis.

Seven candidate miRNAs were selected for expression-level confirmation using stem-loop RT-PCR.  Figure 6 shows that all of the 7 predicted miRNAs were expressed in the *Chiloscyllium plagiosum* liver. With 18s rRNA as positive control, the expression of three target miRNAs was normalized and used to analyze the expression of the miRNA at different liver regenerative stages.  Figure 7 indicates that there is a large transcriptional difference between three known miRNAs at different stages of liver regeneration. Significant miRNA expression was observed in regenerating liver tissues at 6 h and 12 h. The expression level of xtr-miR-125b was the highest in regenerating liver tissues at 6 h and was 40 times higher than that in normal liver tissue. Levels of fru-miR-204, xtr-miR-125b, and has-miR-142-3p_R-1 in regenerating liver tissues at 24 h were 0.685, 0.86, and 1.586 times higher than those in normal liver tissue, respectively (Table 5). This result is consistent with the sequencing data.

### 3.5. Differential Gene Sequence Analysis

**Figure 8:**
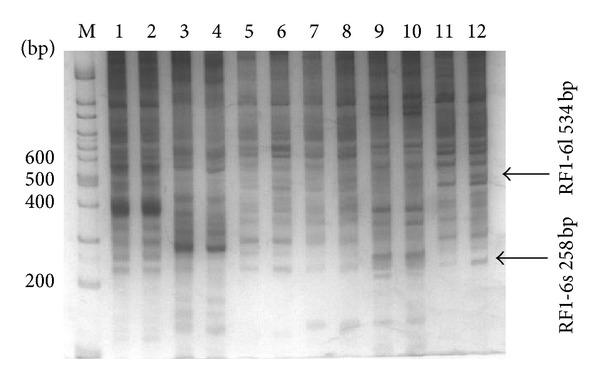
Results of DDRT-PCR. M: 100 bp DNA ladder marker; Lanes 1–12: NF1-1, RF1-1, NF1-2, RF1-2, NF1-3, RF1-3, NF1-4, RF1-4, NF1-5, RF1-5, NF1-6, RF1-6.

Forty pairs of PCR product results were analyzed by 5% polyacrylamide gel electrophoresis and silver staining, and the bands were clear. Six pairs of PCR products are presented in Figure 8, of which two obvious differential bands showed higher expression in regenerating liver. Overall, ten differential sequences were obtained through differential display analysis.

These ten differential sequences were run through BLAST. Three sequences exhibited high similarity to NCBI EST library sequences, and the other 7 sequences had no homology to any known genes. The homology of RF1-6l with 33* Chiloscyllium griseum *cDNA clone sequences was up to 100%. The homology of NF2-2 with 2 cloudy catshark embryo cDNA library sequences was higher than 80%. The homology of RF2-6s with dogfish shark stem cell line sequences was 88%. The matching and differential expression results are presented in Table 6 and consist of 8 sequences ranging from 100 to 500 bp. Three sequences showed high similarity to NCBI EST library sequences through BLAST; 5 sequences were overexpressed, and another five were downregulated during liver regeneration.

## 4. Discussions

The emergence of high-throughput sequencing technology has greatly hastened the discovery of small expressed RNAs in newly analyzed and rare species. Because of the high sequence homology between miRNAs from related species and the stem-loop structure of their precursor sequences, miRNA precursor sequences can be used to perform homology screens covering the entire genome of species. These target gene sequences can be identified using RNA secondary structure analysis software (e.g., RNAfold, MirScan, and MFold) combined with dynamic analysis [35]. A combination of deep sequencing and bioinformatics analysis allows for the identification of new and rarely expressed nonconserved miRNAs, especially in less frequently studied species [36, 37]. 

Here, we report the first complete analysis of miRNA populations in the *Chiloscyllium plagiosum* liver using deep sequencing and bioinformatics analysis. Due to the high conservation of miRNAs between related species, we selected pre-miRNAs/miRNAs of* Danio rerio*,* Fugu rubripes*,* Oryzias latipes*,* Xenopus laevis*,* Xenopus tropicalis*,* Homo sapiens*, and* Mus musculus *to map the reads. In the present study, 257 miRNAs were identified, of which 166 were specific to* Chiloscyllium plagiosum*. These results indicate that the method was effective for identifying low-abundance and species-specific small RNAs. Comparative analysis of the *C*. *milii* genome with the whole-genome assemblies of *Danio rerio*,* Fugu rubripes*, and *Homo sapiens* suggested that (i) noncoding sequences in *Callorhinchus milii* are evolving more slowly than in teleost fishes and that (ii) the *Callorhinchus milii* genome has experienced fewer chromosomal rearrangements compared with teleost fish genomes. Although cartilaginous fish diverged from the human lineage before teleost fish, a higher proportion of regulatory elements are conserved between cartilaginous fish and humans than between teleost fish and humans [16, 38]. Based on conclusions that were drawn from miRNA analysis, our research conclusively demonstrated that cartilaginous and teleost fishes may not have as close a genetic relationship as previously thought. 

In the present study, we observed that the frequency of most PC miRNAs was extremely low. This observation is in accordance with the low expression observed for nonconservative miRNAs and for those involved in organizational or special development periods. Moreover, miRNA genes often form clusters in the genome. Certain miRNAs in genes clusters share the same set of control sequences and are found on the same transcript, whereas some miRNAs in gene clusters are transcribed from independent transcripts, such as miR-433 and miR-127 [39]. An miRNA gene can produce two different miRNAs through duplex transcription and thus control different target genes [40]. A total of 34 miRNA gene clusters were identified in this study, of which 16 produced 32 miRNA groups through duplex transcription. Each of these duplex groups produced 2 miRNAs. This finding provides important clues for probing the function of these miRNAs in future research.

Through miRNA target prediction and GO analysis, we focused on the relation between miRNAs and their targets, thus focusing on mechanisms that involved cell proliferation, immune system processes, and other biological functions. However, as the genome of *Chiloscyllium plagiosum* is not fully sequenced and annotated, it was difficult to determine whether these miRNA targets have any functional bias. It should be noted that many targets could not be annotated in the GO database due to their low homology to orthologs; thus, the miRNA-target network is likely more complex than the results presented here. MiRNA target genes were mostly predicted through bioinformatics software, and miRNA binding sites in most species are in 3′-untranslated regions of the target gene. However, it has been reported that siRNA-mediated translational inhibition can be induced by an incomplete complementary site in the ORF region of a mammalian reporter gene, which means that the target sequence of a given miRNA is not only in the 3′-untranslated region. Because the miRNA and genome of *Chiloscyllium plagiosum *have not been reported and the NCBI database includes just 33 mRNAs, the ESTs and these 33 mRNAs of *Chiloscyllium plagiosum* were used to predict miRNA target genes. The target genes should also be tested using a dual luciferase reporter gene assay system, western blot, or some other experimental method. Through target predictions for miRNAs and GO analysis, we can predict that the target genes are related to thirty-eight biological functions related to “molecular function,” “cellular component,” or “biological process.” A total of 46 related miRNA were screened for cell proliferation and growth, and only eight miRNA have been reported in liver regeneration contexts. Our results will greatly promote research about liver regeneration mechanisms related to miRNA.

Liver regeneration involves regulation of cell proliferation, including such processes as G0/G1 and G1/S transitions of the cell cycle and cell division. Differential expression levels of three miRNAs related to cell proliferation and apoptosis were tested in regenerating liver tissues at 0 h, 6 h, 12 h, and 24 h through the real-time PCR. We determined that three target miRNAs (hsa-miR-142-3p_R-1, xtr-miR-125b, and fru-miR-204) exhibited great differential transcription in regenerating liver tissues at 0 h, 6 h, 12 h, and 24 h with higher expression in the early timepoints of liver regeneration. Expression of miRNAs showed a rising trend in regenerating liver tissues at 6 h, 12 h but in the later timepoints presented a downward trend, with normal levels in regenerating liver tissues at 24 h. It has been reported that apoptotic activity increases significantly in the early timepoints (0–6 h) and late timepoints (4–7 d) of liver regeneration. Therefore, we can speculate that the expression of these three miRNAs increased significantly in the start-up stage of liver regeneration. These miRNAs may be relevant to the inhibition of cell proliferation and DNA synthesis. After 24 h, expression decreased to normal. This may be relevant to the S stage of DNA synthesis, which peaks at this time. In addition, it has been reported that the expression of miRNA showed a rising trend in regenerating liver tissues of ratsat 3 h, 12 h, and 18 h after two-thirds PH, and expression of 70% of miRNAs showed a downward trend in regenerating rat livers 24 h after two-thirds PH. Expression of genes relevant to miRNA generation began to decrease 18–36 h after PH, most obviously 24 h after PH, including RNasen, Dgcr8, Dicer, Tarbp2, and ect [41]. Therefore, we can speculate that this upregulation followed by downregulation of expression may be induced by a negative feedback mechanism involving miRNA generation. It has been reported that miR-142-3p controls the target gene RAC1 and inhibits liver cancer cell proliferation and invasion [42], that miR-204a negatively controls the target gene Bcl-2 to promote apoptosis [43], and that miR-125b was upregulated in Taxol-resistant cells, causing a marked inhibition of Taxol-induced cytotoxicity and apoptosis and a subsequent increase in the resistance to Taxol in cancer cells [44]. Therefore, combining the reported references on miRNAs in liver regeneration and the related signal transduction network references may be helpful to screen and test the liver regeneration miRNAs and their target genes. Researching mechanisms such as when and how the interactions between miRNAs and target genes occur will deepen the understanding of miRNA regulation in the injured liver.

## 5. Conclusions

This study provides the first large-scale identification and characterization of *Chiloscyllium plagiosum* miRNAs, adds a significant number of novel miRNA sequences to the currently available database, and lays the foundation for further understanding of miRNA function in the regulation of *Chiloscyllium plagiosum* liver development. However, considerable work remains to confirm the identity of these miRNAs and their functional significance. Despite the conservation of miRNAs, it is likely that we overlooked several *Chiloscyllium plagiosum* specific miRNAs due to the unavailability of genome sequences for this species. Moreover, the expressed population of miRNAs can change in different tissues and at different development stages. The identified miRNAs may not represent all of the miRNAs that exist in *Chiloscyllium plagiosum*, and more research is required to acquire a full set of miRNAs for this species.

## Supplementary Material

Supplementary Table 1 is about the information on all of miRNAs, which contains the miRNAs' names, sequences, genome IDs, read numbers et al.
Supplementary Table 2 is about the analysis of miRNA gene cluster. It contains 34 clusters in the Callorhinchus milii genome, of which 16 clusters were generated from the 16 pre-miRNAs, and 2 mature miRNAs were cleaved from each pre-miRNA.
Supplementary Table 3 is about the information of miRNA and its target genes related to liver regeneration, which contains forty-six miRNAs were related to cell proliferation and growth terms.

## Figures and Tables

**Table 1:** Criteria used for miRNAs annotation and hairpin structure determination.

miRNAs annotation	
The miR_name is composed of the known miR name in a cluster, an underscore, and a matching annotation such as	
L−*n* means that the miRNAs_seq (detected) is *n* base less than known rep_miRSeq in the left side;	
R−*n* means that the miRNAs_seq (detected) is *n* base less than known rep_miRSeq in the right side;	
L+*n* means that the miRNAs_seq (detected) is *n* base more than known rep_miRSeq in the left side;	
R+*n* means that the miRNAs_seq (detected) is *n* base more than known rep_miRSeq in the right side;	
2ss5TC13TA means 2 substitutin (ss), which are T → C at position 5 and T → A at position 1.	
The miRNAs_seq (detected) is exactly the same as known rep_miRSeq; miRNAs_name is the name of representative miRNAs.	

Hairpin determination	

Definition of MFEI: MFEI = −*dG*∗100/mirLen/CG%.	
Criteria:	
(1) free energy (*dG* in kCal/mol) ≤−15,	
(2) length of hairpin (up and down stem + terminal loop) ≥50,	
(3) number of basepairs (bp) in stem region ≥16,	
(4) length of terminal loop ≤20,	
(5) number of basepairs (bp) in mature or mature ∗ region ≥12,	
(6) percentage of small RNA in stem region (pm) ≥80%,	
(7) number of allowed errors in mature region ≤7,	
(8) number of allowed errors in one bulge in stem ≤12,	
(9) number of allowed errors in one bulge in mature region ≤8,	
(10) number of allowed biased errors in one bulge in mature region ≤4,	
(11) number of allowed biased bulges in mature region ≤2,	
(12) MFEI ≥ 0.7.	

**Table 2:** Primers used in RT-PCR experiment.

Gene	Primer
PC-3p-186_7748-RT	5′-GTCGTATCCAGTGCAGGGTCCGAG GTATTCGCACTGGATACGACATAGGC-3′
PC-5p-108_13860-RT	5′-CTCAACTGGTGTCGTGGAGTCGGC AATTCAGTTGAGAGCTTT-3′
PC-5p-14_74120-RT	5′-CTCAACTGGTGTCGTGGAGTCGGCA ATTCAGTTGAGATTCTC-3′
PC-5p-970_1302-RT	5′-CTCAACTGGTGTCGTGGAGTCGGCAA TTCAGTTGAGGGACAT-3′
hsa-miR-142-3p_R-1-RT	5′-CTCAACTGGTGTCGTGGAGTCGGCA ATTCAGTTGAGCCATAA-3′
fru-miR-126-RT	5′-CTCAACTGAATTGCCGACTCCACGACACCAGTTGAGGCATTA-3′
fru-miR-204-RT	5′-GTCGTATCCAGTGCAGGGTCCGAGG TATTCGCACTGGATACGACATGCCT-3′
PC-3p-186_7748-Forward	5′-GCACTTGTCCCAGCCTATGTC-3′
PC-5p-108_13860-Forward	5′-CCGTGTAAACATCTGTAACTGAAAG-3′
PC-5p-14_74120-Forward	5′-GCGAGCACTGTCTAACCTGAG-3′
PC-5p-970_1302-Forward	5′-GGTCCAACCTCTTATGTCCCTC-3′
hsa-miR-142-3p_R-1-Forward	5′-GGCTTTAGTGTTTCCTACTTTATGG-3′
fru-miR-126-Forward	5′-TCCTACCGTGAGTAATAATGCC-3′
fru-miR-204-Forward	5′-CGTGTTCCCTTTGTCATCCTATC-3′
18s rRNA-Forward	5′-GACTCAACACGGGAAACCTCA-3′
18s rRNA-Reverse	5′-CAGACAAATCGCTCCACCAA-3′
miR-Reverse1	5′-AGCAGGGTCCGAGGTATTC-3′
miR-Reverse2	5′-GTGTCGTGGAGTCGGCAAT-3′
miR-Reverse3	5′-AACTGAATTGCCGACTCCAC-3′

**Table 3:** The primers of DDRT-PCR.

Primer	Sequence	Primer	Sequence
Anchor primer 1	5′-AGCTTTTTTTTTTTVA-3′	Anchor primer 2	5′-AGCTTTTTTTTTTTVC-3′
Random primer 1	5′-GCTAACGATG-3′	Random primer 2	5′-TGGATTGGTC-3′
Random primer 3	5′-CTTTCTACCG-3′	Random primer 4	5′-TTTTGGCTCC-3′
Random primer 5	5′-GGAACCAATG-3′	Random primer 6	5′-AAACTCCGTC-3′
Random primer 7	5′-TCGATACAGG-3′	Random primer 8	5′-TGGTAAAGGG-3′
Random primer 9	5′-TCGGTCATAG-3′	Random primer 10	5′-CTGCTTGATG-3′
Random primer 11	5′-TACCTAAGCG-3′	Random primer 12	5′-CTGCTTGATG-3′
Random primer 13	5′-GTTTTCGCAG-3′	Random primer 14	5′-GATCAAGTCC-3′
Random primer 15	5′-GATCCAGTAC-3′	Random primer 16	5′-GCTCACGTAG-3′
Random primer 17	5′-GATCTGACAC-3′	Random primer 18	5′-GCTATCAGAC-3′
Random primer 19	5′-GATCATAGCG-3′	Random primer 20	5′-GATCAATCGC-3′

**Table 4:** Distribution of small RNA among LN and LR24.

Category	Total RNAs in LN	Percent	Total RNAs in LR24	Percent
Total small RNAs	15361801	100%	10146583	100%
Group 1a	0	0.00%	0	0.00%
Group 1b	1600835	10.40%	985411	9.70%
Group 2a	56020	0.40%	39787	0.40%
Group 2b	25388	0.20%	12569	0.10%
Group 3a	2300528	15.00%	1113914	11.00%
Group 3b	72334	0.50%	40956	0.40%
Group 4a	310150	2.00%	153216	1.50%
Group 4b	301238	2.00%	195545	1.90%
Group 5	6032607	39.30%	4341986	42.80%
Group 6	4662701	30.40%	3263199	32.20%

**Table 5:** Relative quantitative data analysis of miRNA in four periods regenerative liver tissues.

Gene	Average *C*_*t*_ value ± standard error				2^−ΔΔ*C*_*t*_^			
	LN	LR6	LR12	LR24	LN	LR6	LR12	LR24
18S rRNA	21.982 ± 0.116	21.982 ± 0.116	23.160 ± 0.016	23.267 ± 0.127	1.000	1.000	1.000	1.000
xtr-miR-125b	25.710 ± 0.141	25.710 ± 0.141	22.637 ± 0.354	26.400 ± 0.222	1.000	43.070	3.676	0.860
has-miR-142-3p_R-1	21.035 ± 0.265	21.035 ± 0.265	21.126 ± 0.239	21.395 ± 0.202	1.000	4.803	4.623	1.586
fru-miR-204a	18.316 ± 0.383	18.316 ± 0.383	18.598 ± 0.122	19.730 ± 0.135	1.000	4.209	2.226	0.685

**Table 6:** Analysis of differential expressed sequences.

Clone	Length (bp)	Query coverage	Max identity to the EST
RF1-6l	534	11%	100%
RF1-6s	258		novel
RF2-8	283		novel
NF2-2	173	94%	80+%
NF1-7	236		novel
NF1-19	326		novel
RF2-6s	244	78%	88%
RF2-6l	278		novel
NF2-19	77		novel
NF1-9	208		novel
